# Kidney‐sparing surgery for distal high‐risk ureteral carcinoma: Clinical efficacy and preliminary experiences in 22 patients

**DOI:** 10.1002/cam4.5544

**Published:** 2022-12-19

**Authors:** Yu Jiang, Yueqiang Peng, Siwei Ding, Yongbo Zheng, Yunfeng He, Jiayu Liu

**Affiliations:** ^1^ Department of Urology The First Affiliated Hospital of Chongqing Medical University Chongqing China

**Keywords:** kidney‐sparing, living quality, risk stratification, surgery, ureter cancer

## Abstract

**Background:**

Several groups proved kidney‐sparing surgery (KSS) had equivalent oncological outcomes compared with radical nephroureterectomy (RNU) for the low‐risk upper urinary tract urothelial carcinoma (UTUC) patients. Whereas, the clinical efficacy of KSS for high‐risk UTUC, especially for distal high‐risk ureteral carcinoma, remains unclear.

**Objective:**

To evaluate the feasibility of KSS for patients with distal high‐risk ureter cancer.

**Materials and methods:**

Our study included 22 patients who diagnose the distal high‐risk ureter cancer and underwent KSS between May 2012 and July 2021 in the First Affiliated Hospital of Chongqing Medical University. Overall survival (OS), confirmed as the primary endpoint of present study, was assessed by a blinded independent review committee (BIRC). The secondary endpoints included the postoperative SF‐36 (the short form 36 health survey questionnaire) score, progression‐free survival (PFS), postoperative complications, and so on.

**Results:**

Overall, 17 (77.3%) and 5 (22.7%) patients underwent segmental ureterectomy (SU) and endoscopic ablation (EA), respectively. By the cut‐off date, the mean OS was 76.3 months (95% Cl: 51.3–101.1 months) and the mean PFS was 47.0 months (95% Cl: 31.1–62.8 months), respectively. And the SF‐36 score in a majority of patients was >300 (90.9%).

**Conclusion:**

This is a daring endeavor to explore the clinical efficacy of KSS in distal high‐risk ureter cancer based on the high‐risk UTUC criteria, which shows satisfactory results in the long‐term prognosis and operation‐associated outcomes. However, future randomized or prospective multicenter studies are necessary to validate our conclusions.

## Graphical abstract

We explored the clinical efficacy of kidney‐sparing surgery (KSS) in distal high‐risk ureter cancer based on the high‐risk UTUC criteria, which was a daring endeavor. In present study, the long‐term prognosis and operation‐associated outcomes were all satisfactory, even in patients with relatively advanced TNM stage. Future randomized or prospective multicenter large‐scale researches remains necessary for further validation.

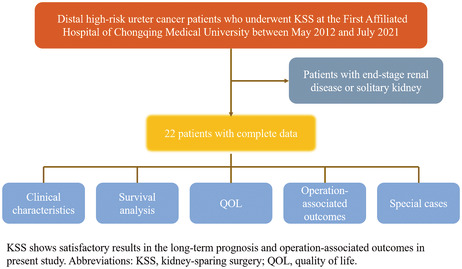

## 1 INTRODUCTION

**FIGURE 1:**
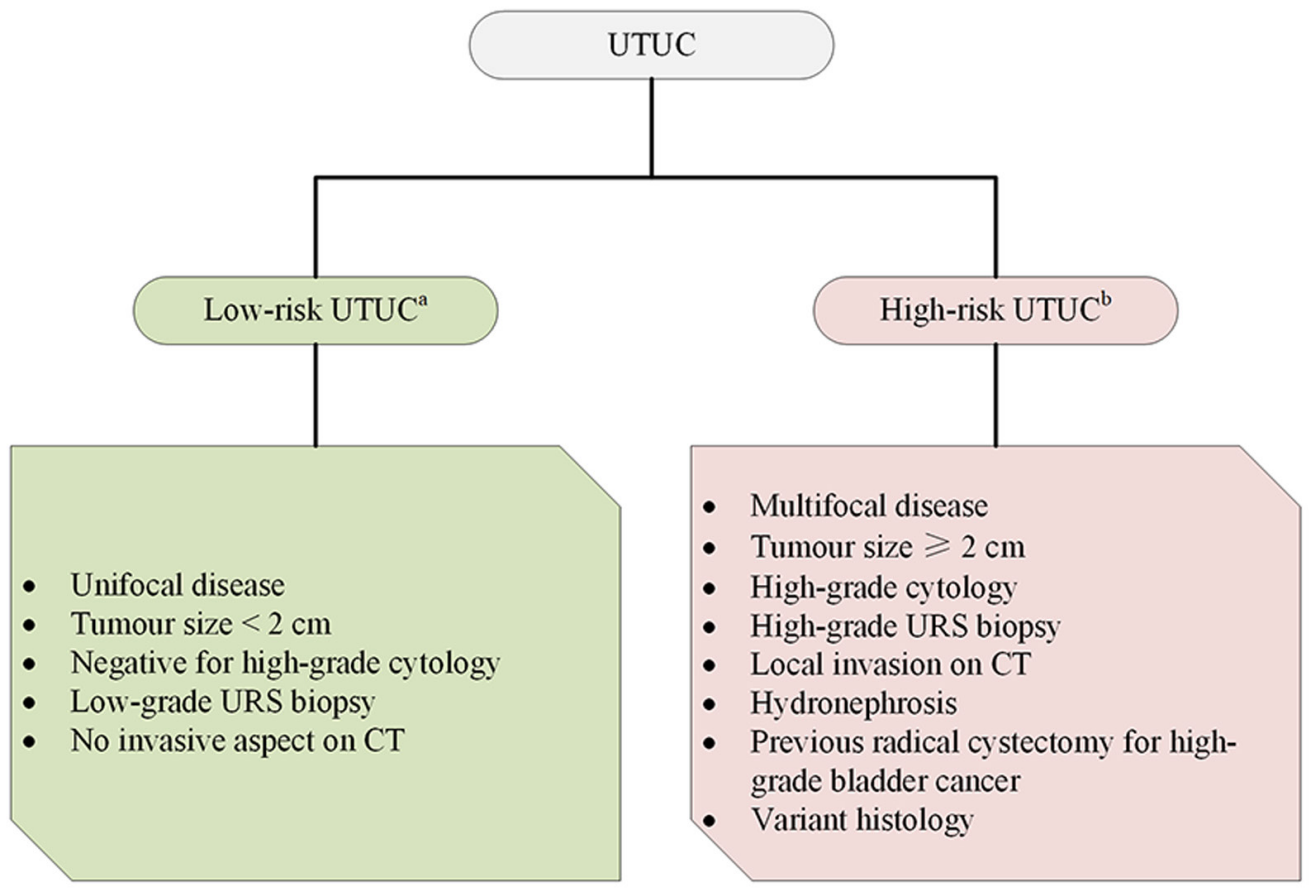
The risk stratification for UTUC patients which admitted by EAU guidelines. (A) All these factors need to be present. (B) Any of these factors need to be present. CT, computed tomography; URS, ureteroscopy; UTUC, upper urinary tract urothelial carcinoma.

Ureteral cancer is a relatively rare tumor of the urinary system. Upper urinary tract urothelial carcinoma (UTUC) accounts for approximately 5–10% of urothelial cancers, while ureteral cancer represents only a third.^1^ And ureter cancer usually means poor prognosis and life quality. For purpose of making a decision on the most appropriate treatment and discussing systemic therapy preoperatively, urological surgeons are used to risk‐stratify patients based on so many identified prognostic factors of UTUC and several risk stratification models were reported,^2^, ^3^, ^4^ one of which was recognized by European Association of Urology (EAU) (Figure 1).^5^ On the basis of the EAU criterion, those who suffered from multifocal, large, high‐grade, invasive diseases or had ipsilateral hydronephrosis, bladder cancer (BCa) histories, variant histology were defined as high‐risk UCUC. To date, nephroureterectomy (RNU) still proves to be the primary care for patients with high‐risk nonmetastatic UTUC. Although feasibility of KSS for high‐risk ureter cancer needs confirmation, increasing positive evidences have demonstrated the benefits of kidney‐sparing surgery (KSS), including slighter surgical trauma, lower complication rate, earlier hospital discharge, and less hospitalization expenses.

KSS, known as a series of surgery, includes ureteroscopy, ureteral resection, and so on. Several researchers have demonstrated patients with low‐risk UTUC undergoing KSS showed a similar survival to who undergoing RNU. Thomas S et al. reported a large‐sample study recruiting 2,299 patients which showed KSS, comparing with RNU, for low‐risk UTUC reduced the morbidity such as renal dysfunction, without sacrificing oncological outcomes.^6^ In comparison with RNU, fewer cardiovascular‐related events occurred when KSS was underwent.^7^ Therefore, KSS became a preferred approach for low‐risk UTUC according to EAU guidelines (version 2021). Moreover, RNU, which has remained the primary treatment strategy for UTUC, had no significant breakout in tumor outcomes in the past three decades, especially the improvement of living quality. Jonathan L et al. analyzed data for 120 patients collected in Memorial Sloan‐Kettering Cancer Center, revealing similar oncologic outcomes between patients treated with KSS and RNU, while more renal function losses were observed in patients underwent RNU.^8^

Although KSS is not preferred for high‐risk ureter cancer in each guideline, in daily clinical work, some patients with high‐risk ureter cancer and relatively poor physical fitness desire KSS, largely due to the slighter adverse reaction of postoperative adjuvant chemotherapy and less complications after surgery. Accordingly, KSS has been gaining more and more attention in the evaluation before surgery. And we found that the patients underwent KSS had well life qualities, with a similar survival to those underwent RNU. Meanwhile, more hemorrhage and longer operation time of RNU,^9^ which is non‐negligible, also made a contribution to the patients' choices of KSS when we communicate with them preoperatively, although we emphasized the primary standard approach of UTUC and the risk of KSS. Therefore, we considered whether KSS could be performed in distal high‐risk patients, in view of several patients' intolerance to RNU, when the surgeon was sophisticated enough, and whether patients with certain clinical characteristics would have a similar or even better prognosis after KSS compared with RNU. To the best of our knowledge, a few articles reporting patients who underwent segmental ureterectomy (SU), simply using individual criteria to recruit the high‐risk cohort, had mixed results.^10^ The inclusion criteria of high‐risk cohorts in those articles were all incomplete. Thus, for high‐risk UTUC, especially distal high‐risk ureter cancer, it remains uncertain to estimate the feasibility of KSS.

In summary, we conducted this study to assess the safety and feasibility of KSS in distal high‐risk ureter cancer patients. And we wished that present study could provide a reference for the selection of patients better suited to undergo KSS.

## 2 MATERIALS AND METHODS

### 2.1 Patient selection

The study was approved by the Institutional Ethics Board of the First Affiliated Hospital of Chongqing Medical University. We retrospectively collected data on patients who underwent KSS at the First Affiliated Hospital of Chongqing Medical University between May 2012 and July 2021. Diagnosis of ureter cancer could be confirmed by biopsy, cytology with a mass on cross‐sectional imaging, or cytology with ureteroscopic mass visualization. All the patients were assessed as high‐risk UTUC according to EAU guidelines (version 2021). Patients who followed up less than 6 months or those receiving neoadjuvant therapies were excluded. Meanwhile, the ones with end‐stage renal disease or solitary kidney were also counted out.

In our study, the clinicopathologic characters, collected from the computerized medical records, of all the participants were retrospectively reviewed. The study was conducted in compliance with the Declaration of Helsinki and Good Clinical Practice guidelines. All data were anonymized before accession and thus the requirement for informed consent was waived by the ethical committee, considering the retrospective nature of this study.

### 2.2 Procedure

SU was performed according to the standard criteria: extrafascial dissection of the lesion with wide margins and complete bladder cuff excision. Complete tumor resection or destruction was assessed by individual surgeons in the EA. Specifically, in our medical center, all the KSSs were undergone in distal ureter cancer in consideration of the increasing operative difficulty and risk in upper ureter cancer. Most importantly, surgeons stress emphasized the primary standard therapy of high‐risk UTUC. Each patient was informed of the risk of recurrence and those underwent EA were informed that it's of vital importance to conduct an early second‐look ureteroscopic and stringent surveillance. All patients followed up every 12 weeks after surgery. Otherwise, the relations would receive a follow‐up call. Blood losses were estimated during operation by the surgeon. All postoperative complications were monitored and graded according to the Clavien–Dindo classification.^11^ The quality of life (QOL) was assessed according to the Eastern Cooperative Oncology Group (ECOG) score and SF‐36 (the short form 36 health survey questionnaire) score, which were confirmed by medical records and telephone follow‐up.

### 2.3 Statistical analysis

All reported *p* values are two‐sided and statistical significance was set at <0.05. The Kaplan–Meier estimate was employed to calculate and plot survival curves. Our primary clinical outcome was overall survival (OS) which was assessed by the blinded independent review committee (BIRC). The secondary endpoints included QOL, progression‐free survival (PFS), and postoperative complications. All analyses were performed with SPSS 25.0 software.

## 3 RESULTS

### 3.1 Clinical characteristics

Between May 2012 and July 2021, 22 KSSs were performed, of which 17 (77.3%) were SU while 5 (22.7%) were EA, including 15 (68.2%) male and 7 (31.8%) female patients. The median age was 68 years (range: 38–91 years). At baseline, most patients (72.7%) showed hydronephrosis on computerized tomography (CT), and 17 (77.3%) were demonstrated high‐grade pathology. Four patients had BCa histories, which were all diagnosed as high‐grade UC and had undergone transurethral resection of the bladder (TURB). Four (18.2%) patients underwent cystoscopies while other six (27.3%) patients underwent ureteroscopies before surgery in order to confirm the feasibility of KSSs. None of the participants suffered from upper or middle ureter cancer. Demographic and perioperative parameters are listed in Table 1.

**TABLE 1:** Demographic and clinical characteristics of 22 patients treated with KSS due to high‐risk ureter cancer

Characteristics	Treated patients with KSS, total (*n* = 22)
Age, years	
Median	68
Mean ± SD	69.0 ± 11.0
P25, P75	64, 76
BMI, kg/m^2^	
Median	23.71
Mean ± SD	23.6 ± 3.5
P_25_, P_75_	21.33, 25
Gender, *n* (%)	
Female	7 (31.8%)
Male	15 (68.2%)
Lateral, *n* (%)	
Right	12 (54.6%)
Left	10 (45.4%)
Location, *n* (%)	
Distal ureter	13 (59.1%)
Ureteral orifice	9 (40.9%)
Scopy before operation, *n* (%)	
Never	12 (54.6%)
Ureteroscopy	6 (27.3%)
Cystoscopy	4 (18.2%)
Tumor size on CT, *n* (%)	
< 2 cm	9 (40.9%)
≥ 2 cm	13 (59.1%)
Basic diseases, *n* (%)	
Hypertension	7 (31.8%)
Diabetes	4 (18.2%)
Coronary heart disease	0
Preoperative GFR, ml/(min*1.73m^2^)	
Median	74.2
Mean ± SD	65.5 ± 5.3
P25, P75	42.4, 80.3
Complication, *n* (%)	
Hematuria	9 (40.9%)
Pain	9 (40.9%)
Treatment after surgery, *n* (%)	
Bladder instillation	16 (72.7%)
Adjuvant radiotherapy	4 (18.2%)
History of BCa, *n* (%)	4 (18.2%)
Hydronephrosis on CT, *n* (%)	16 (72.7%)
Smoking history, *n* (%)	6 (27.3%)
Type of surgery, *n* (%)	
SU	17 (77.3%)
EA	5 (22.7%)

Abbreviations: BCa, bladder cancer; BMI, body mass index; CT, computerized tomography; EA, endoscopic ablation; GFR, glomerular filtration rate; SU, segmental ureterectomy.

### 3.2 OS and PFS

**FIGURE 2:**
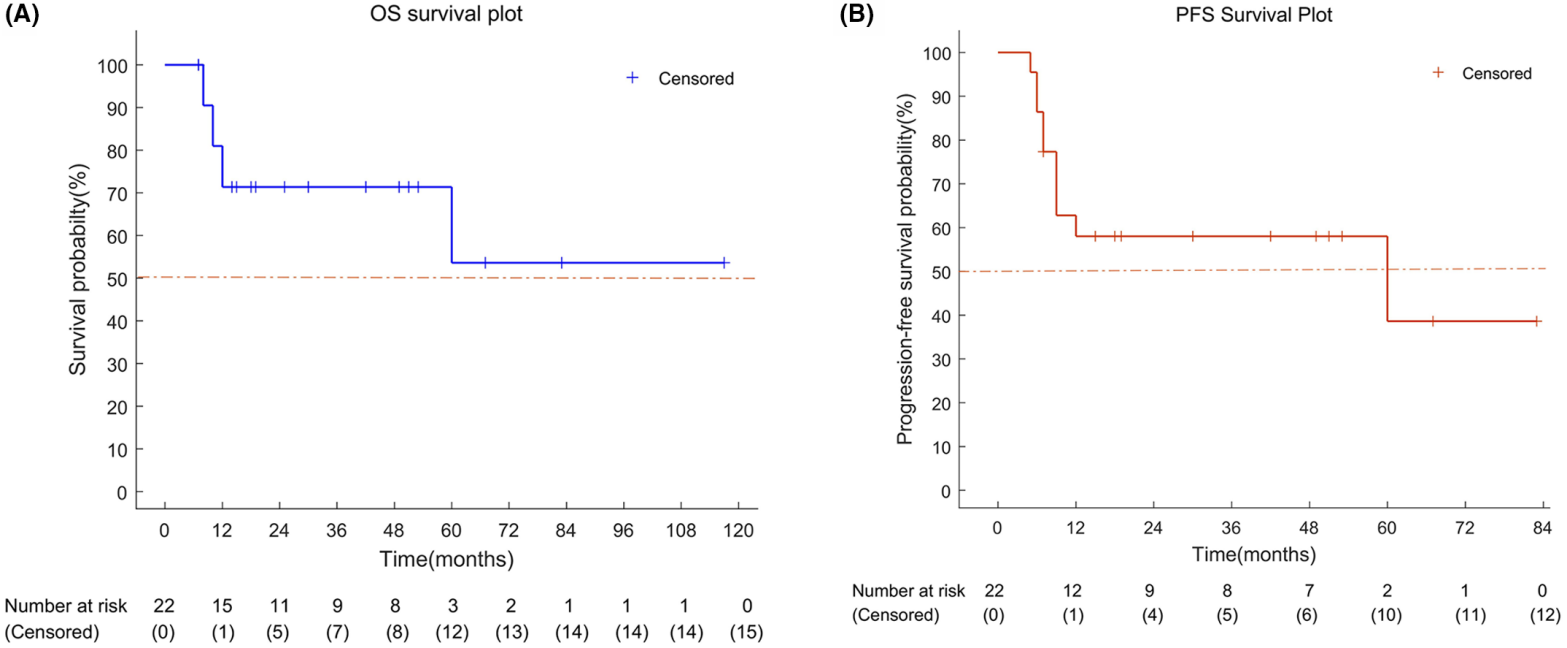
(A) Kaplan–Meier estimates of OS as assessed by the BIRC. (B) Kaplan–Meier estimates of PFS.

**FIGURE 3:**
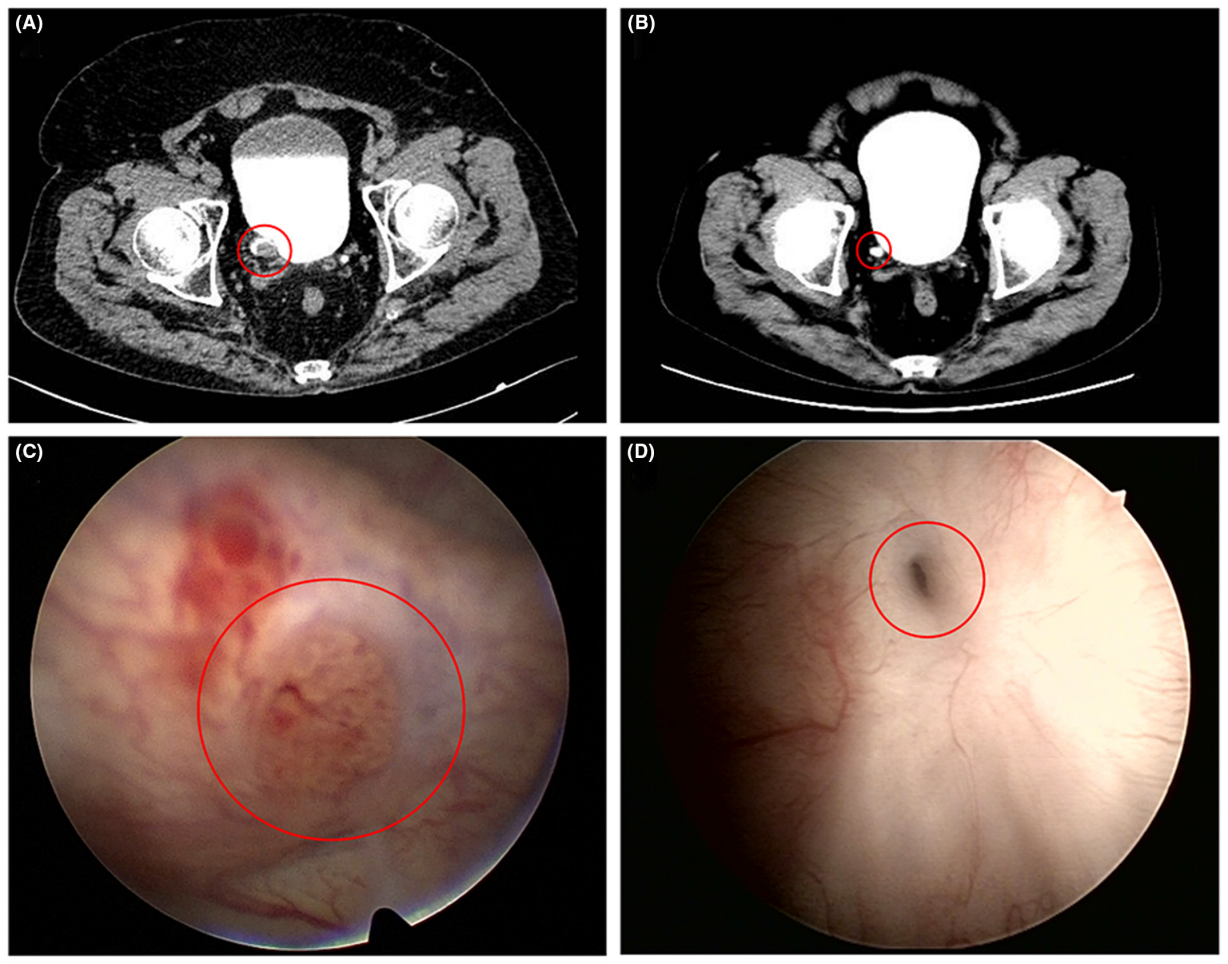
The lesion location was circled in red. (A) The preoperative computed tomography urography (CTU) of a patient who underwent EA. (B) The postoperative CTU, which was performed 21 months after the surgery, of the same patient. (C) The preoperative cystoscopy of the same patient. (D) The cystoscopy, which was performed 21 months after the surgery, of the same patient. The approximately same location of the ureter tumor was marked on the illustration.

By the cut‐off date of February 10, 2022, the mean OS was 76.3 months (95% Cl: 51.3–101.1 months) and the mean PFS was 47.0 months (95% Cl: 31.1–62.8 months), respectively. The median PFS was 60 months (95% Cl: 0.0–144.3 months) while the median OS was not reached (Figure 2). In addition, the 5‐year OS rate was 53.6% and the 5‐year PFS rate was 38.6%. Interestingly, the patients who underwent EA have a satisfactory prognosis. The preoperative and follow‐up images are showed in Figure 3.

### 3.3 Quality of life

Preoperatively, ECOG sore was 0 in 2 patients (9.1%), 1 in 11 patients (50.0%), 2 in 7 patients (31.8%), and 4 in 2 patient (9.1%). Meanwhile, the SF‐36 score in a majority of patients was >300 (90.9%). The QOL and the adverse events were listed in Table 2.

**TABLE 2:** The postoperative QOL and the adverse events of all the KSS patients

	Treated patients with KSS, total (*n* = 22)	
	Preoperative	Postoperative
ECOG score^a^		
0	2 (9.1%)	2 (9.1%)
1	13 (59.1%)	11 (50%)
2	5 (22.7%)	7 (31.8%)
3	2 (9.1%)	0
4	0	2 (9.1%)
5	0	0
SF‐36 score^a^		
0~99		2 (9.1%)
100~299		0
300~499		9 (40.9%)
500~		11 (50%)
Median		499.89
Mean ± SD		494.2 ± 187.1
Min, max		388.7, 634.4
Adverse events^b^		
Hematuria		8 (36.4%)
Pain		2 (9.1%)
Urinary frequency		11 (50%)

Abbreviations: ECOG, Eastern Cooperative Oncology Group; SF‐36, the short form 36 health survey questionnaire.

^a^ Collected 6 months after surgery.

^b^ Collected 1 month after surgery.

### 3.4 Operation‐associated outcomes

The mean operative time was 101.9 ± 47.8 min, and the median estimated blood loss was 50.0 ml (range: 10.0–360.0 ml). Mean hospitalization duration was 8.9 ± 7.0 days, where none of the patients was transferred to the intensive care. The major intraoperative complications were intestinal injuries and major vascular injuries, each seen in one patient (total three patients). Pathologic and operation‐associated outcomes are given in Table 3.

**TABLE 3:** The pathologic and operation‐associated outcomes

	Treated patients with KSS, total (*n* = 22)
T stage, *n* (%)	
T1	12 (54.6%)
T2	6 (27.3%)
T3	3 (13.6%)
T4	1 (4.5%)
N stage, *n* (%)	
N0	19 (86.4%)
N1	1 (4.5%)
N2	2 (9.1%)
Pathological grade, *n* (%)	
Low	5 (22.7%)
High	17 (77.3%)
Estimated blood loss (during surgery) (ml)	
Median	50
Mean ± SD	107.3 ± 102.0
P_25_, P_75_	45, 162.5
Operation time (min)	
Median	102.5
Mean ± SD	101.9 ± 47.8
P_25_, P_75_	68.75, 128.75
Length of hospital stay (days)	
Median	7
Mean ± SD	8.9 ± 7.0
P_25_, P_75_	4.5, 11.25
Complication grade, *n* (%)	
Clavien I	6 (27.3%)
Clavien II	1 (4.5%)
Clavien III	0
Clavien IV	0
Clavien V	0

Intuitively, the brief characters and outcomes were showed in Table 4. What is worth mentioning, the N stage of SU was determined by postoperative pathology. But the N stage of EA was determined by imaging diagnosis because, we could not obtain pathological stage during endoscopic ablation. So, we recorded the N stage of EA in the Table 4 as Nx.

**TABLE 4:** Brief characters and outcomes of each patient

ID	Age	Gender	Stage	Surgery	History of Bca	Preoperative ECOG score	Treatment after surgery	Statement	Duration (months)	Postoperative SF‐36
1	71	Male	T_1_NxM_0_	EA	No	0	Intravesical chemotherapy	Alive	19	749
2	68	Male	T_1_NxM_0_	EA	No	2	Neither	Dead	8	549.11
3	64	Female	T_1_N_0_M_0_	SU	No	1	Intravesical chemotherapy	Alive	18	575.78
4	53	Male	T_1_NxM_0_	EA	Yes	0	Intravesical chemotherapy	Alive	25	669.89
5	68	Male	T_2_N_2_M_0_	SU	No	1	Intravesical chemotherapy	Alive	51	708.89
6	70	Male	T_1_N_0_M_0_	SU	No	1	Intravesical and intravenous chemotherapy	Dead	10	358.22
7	91	Male	T_3_N_0_M_0_	SU	No	3	Intravesical and intravenous chemotherapy	Dead	12	66.11
8	70	Male	T_3_N_0_M_0_	SU	No	1	Intravesical chemotherapy	Alive	83	470.44
9	62	Female	T_2_N_0_M_0_	SU	Yes	1	Neither	Alive	67	518
10	82	Male	T_1_N_0_M_0_	SU	No	1	Intravesical chemotherapy	Dead	60	470.11
11	79	Female	T_1_N_0_M_0_	SU	No	2	Neither	Alive	49	395.78
12	81	Female	T_1_N_0_M_0_	SU	No	2	Intravesical chemotherapy	Alive	49	395.78
13	74	Male	T_1_N_0_M_0_	SU	No	1	Neither	Alive	42	331.56
14	67	Male	T_1_N_0_M_0_	SU	No	2	Neither	Alive	117	411.11
15	75	Male	T_2_N_1_M_0_	SU	Yes	2	Intravesical and intravenous chemotherapy	Dead	12	367.56
16	65	Male	T_3_N_2_M_0_	SU	No	3	Neither	Dead	8	90.33
17	38	Male	T_4_N_0_M_0_	SU	No	1	Intravesical chemotherapy	Alive	30	481.78
18	64	Female	T_1_NxM_0_	EA	Yes	1	Intravesical chemotherapy	Alive	53	767.89
19	81	Male	T_2_N_0_M_0_	SU	No	1	Intravesical chemotherapy	Dead	10	588.33
20	60	Female	T_2_N_0_M_0_	SU	No	1	Intravesical and intravenous chemotherapy	Alive	14	622.56
21	67	Male	T_2_N_0_M_0_	SU	No	1	Intravesical chemotherapy	Alive	7	597.11
22	67	Female	T_2_NxM_0_	EA	Yes	1	Intravesical chemotherapy	Alive	15	687.33

## 4 DISCUSSION

Compared with RNU, which excises the entire section of ureter and the ipsilateral kidney, KSS straightly removes or extirpates the tumor and retains the ipsilateral kidney. As we all know, it's a broad trend to improve minimally invasive operation for surgical diseases. And increasingly patients demand of KSS because of psychological factors or other reasons. Importantly, based on less loss of kidney function, KSS might become an alternative on account of it providing more choices for subsequent care such as probably postoperative adjuvant chemotherapy. Besides, continually updated disease risk stratification also led to a favorable rise in KSS for selected patients like those with localized, unifocal, and distal ureter tumors.

KSS and RNU, in patients with low‐risk UTUC, showed comparable oncologic outcomes, which was proved in several articles. Simhan J et al. evaluated 1,227 patients with low‐ or moderate‐grade, localized non‐invasive UTUC, which showed similar cancer‐specific mortality rates between RNU group and KSS group.^12^ In addition, two researches demonstrated endoscopic resection was an acceptable option for those with low grade UTUC.^13^, ^14^ A multi‐institutional retrospective review of 468 participants analyzed recurrence‐free survival, metastasis‐free survival, and cancer‐specific survival. The results showed that there's no significant difference, on univariate analysis, between the two cohorts (SU vs. RNU) on parameters mentioned above.^15^ Similarly, two large‐sample‐size studies demonstrated parallel outcomes between RNU and SU.^16^, ^17^ These findings suggested the feasibility of KSS to patients with appropriate selection, but no reported investigations of KSS based on the recent risk stratification.

In our study, KSS showed a median PFS of 60 months and a mean OS of over 6 years, not worse than that of patients underwent RNU in most of articles. Based on our experience, maybe distal ureter cancer, relatively small sized tumor, and no distant metastasis gave the main assists. And we collected nearly all the high‐risk ureter cancer patients in our medicine center, who had undergone KSS, according to the EAU guidelines without excessive exclusive criteria, avoiding missing any high‐risk patients. Thus, our study is still valuable for analyzing the feasibility of KSS among distal high‐risk patients.

Regrettably, the rate of bladder recurrence is 22–47%, even though undergoing RNU for UTUC.^18^, ^19^ A postoperative intravesical instillation of drugs was demonstrated in risk reduction of bladder tumor recurrence reported in two prospective randomized trials^20^, ^21^ and two meta‐analyses.^22^, ^23^ In present study, by the cut‐off date, 4 (18.2%) patients, followed up for 8 months at least while 117 months at most and 3 of whom were still alive, relapsed in the bladder. Several patients rejected postoperative bladder instillation on account of the poor compliance, but they had a favorable survival of >3 years and still alive until the cut‐off date, except two patients who had reached stage N2.

SF‐36 sore was rarely reported in researches for KSS even in those for low‐risk ureter cancer. Almost all of the participants have a score of >300, which were similar or even better than those before operation. Surprisingly, a patient whose preoperative ECOG was 1 and tumor confined within the mucosal layer had a SF‐36 score of 767.9. In current clinical practice, KSS brought satisfactory QOL to most of the patients, even though to the patients that had disease relapses within 6 months. Nearly half of them were willing to undergo adjuvant chemotherapy and all of them survived over 12 months except a stage‐T3 patient. Furthermore, KSS had showed consistent benefit to the QOL across all the subgroups, including patients with recurrence or metastasis. Of the 8 patients who had determined disease progression, only 1 patient had a relatively lower SF‐36 score less than 300, probably because of the pre‐operative poor ECOG of 3 and the relatively advanced TNM staging(T3N2M0). For this patient, who had a preoperative ECOG score of 3, the families of that patient chose KSS even though we informed them the lymph node metastases during the operation, large due to the probable severe complications of RNU.

KSS was well tolerated in our study. Only one patient suffered from Clavien II complications which were treated by total parenteral nutrition. And more than half of the patients were discharged within 1 week. At 1 month after surgery, 11 patients complained of frequent urination, e8 of them complained of gross hematuria, and 2 of them complained of occasional pain. After further investigation of the case data, 8 of the 11 patients with frequent urination were all male patients over 60 years old, with full prostate on pre‐operative CT, so the symptoms of frequent urination may be associated with prostate hyperplasia. Among the eight patients with gross hematuria, two still had intermittently gross hematuria after 3 months, which was considered to be due to their advanced staging (T3N2M0 and T3N0M0, respectively), larger wound area in the surgical area and older age. This single‐arm study did not compare KSS with RNU because nearly all the patients with localized distal ureter tumor underwent KSS. And due to the lack of cases with ureteral cancer in the particular location, the sample size was too small to demonstrate the superiority of KSS exactly. Besides, this study based on Chinese resident have not explored therapeutic effects by ethnicity or race.

## 5 CONCLUSION

In conclusion, we conducted daring endeavors in KSS for distal high‐risk ureter cancer. In present study, KSS performed well in the long‐term prognosis and operation‐associated outcomes. The KSS cohort had a satisfactory QOL without compromising oncological outcomes, even among the patients with relatively advanced staging, indicating that KSS has the potential to become a treatment option for several selected patients with distal high‐risk distal ureter cancer. However, whether KSS is appropriate for upper ureter cancer is still a controversial problem. And KSS needs to be carried out in patients with high compliance due to the possible underlying risks. Future randomized or prospective multicenter large‐scale researches are necessary for validation of the value of KSS for patients with high‐risk ureter cancer.

## AUTHOR CONTRIBUTIONS

**Yu Jiang:** Conceptualization (equal); formal analysis (lead); methodology (equal); software (lead); writing – original draft (lead); writing – review and editing (equal). **Yueqiang Peng:** Data curation (lead); investigation (equal); validation (lead); writing – review and editing (equal). **Siwei Ding:** Investigation (equal); visualization (equal); writing – review and editing (equal). **Yongbo Zheng:** Methodology (equal); supervision (equal); visualization (equal); writing – review and editing (equal). **Yunfeng He:** Project administration (equal). **Jiayu Liu:** Conceptualization (lead); funding acquisition (lead); methodology (equal); project administration (equal); supervision (equal).

## CONFLICT OF INTEREST

The authors declare that they have no competing interest.

## Data Availability

The data that support the findings of this study are available from the corresponding author upon reasonable request.
